# A Modeled Comparison of Direct and Food Web-Mediated Impacts of Common Pesticides on Pacific Salmon

**DOI:** 10.1371/journal.pone.0092436

**Published:** 2014-03-31

**Authors:** Kate H. Macneale, Julann A. Spromberg, David H. Baldwin, Nathaniel L. Scholz

**Affiliations:** Northwest Fisheries Science Center, National Marine Fisheries Service, National Oceanic and Atmospheric Administration, Seattle, Washington, United States of America; Texas Tech University, United States of America

## Abstract

In the western United States, pesticides used in agricultural and urban areas are often detected in streams and rivers that support threatened and endangered Pacific salmon. Although concentrations are rarely high enough to cause direct salmon mortality, they can reach levels sufficient to impair juvenile feeding behavior and limit macroinvertebrate prey abundance. This raises the possibility of direct adverse effects on juvenile salmon health in tandem with indirect effects on salmon growth as a consequence of reduced prey abundance. We modeled the growth of ocean-type Chinook salmon (*Oncorhynchus tshawytscha*) at the individual and population scales, investigating insecticides that differ in how long they impair salmon feeding behavior and in how toxic they are to salmon compared to macroinvertebrates. The relative importance of these direct vs. indirect effects depends both on how quickly salmon can recover and on the relative toxicity of an insecticide to salmon and their prey. Model simulations indicate that when exposed to a long-acting organophosphate insecticide that is highly toxic to salmon and invertebrates (e.g., chlorpyrifos), the long-lasting effect on salmon feeding behavior drives the reduction in salmon population growth with reductions in prey abundance having little additional impact. When exposed to short-acting carbamate insecticides at concentrations that salmon recover from quickly but are lethal to invertebrates (e.g., carbaryl), the impacts on salmon populations are due primarily to reductions in their prey. For pesticides like carbaryl, prey sensitivity and how quickly the prey community can recover are particularly important in determining the magnitude of impact on their predators. In considering both indirect and direct effects, we develop a better understanding of potential impacts of a chemical stressor on an endangered species and identify data gaps (e.g., prey recovery rates) that contribute uncertainty to these assessments.

## Introduction

Throughout California and the Pacific Northwest, pesticides are frequently detected in aquatic habitats that support threatened and endangered Pacific salmon (*Oncorhynchus* spp.) [1], [2]. Whether these pesticides pose a risk to salmon depends on multiple factors, including the biochemical properties of the pesticides and the effects they have on salmon physiology. For instance, different pesticides that inhibit the same critical enzyme in juvenile salmon can vary in their long-term effects. Such is the case for two classes of insecticides, carbamates and organophosphates, which differ in how long they reduce a salmon’s ability to swim and feed normally. Both classes inhibit acetylcholinesterase (AChE), an enzyme required for the proper functioning of cholinergic synapses in vertebrate and invertebrates. Sublethal exposures of AChE-inhibiting insecticides can cause juvenile salmon to feed less [3], [4] and swim irregularly [5]–[7]. These effects may persist for only a few hours after an exposure if the insecticide is a carbamate [8], yet it may take many weeks to months for a fish to resume normal feeding after exposure to an organophosphate [9], [10]. Although a difference in feeding recovery time may seem subtle when evaluating toxicological effects, a prolonged reduction in feeding can affect the growth and survival of juveniles and ultimately impact the population [11]. Reduced growth is especially critical for juvenile salmon, because smaller fish have lower first-year survival [12]–[14].

How quickly salmon resume normal feeding is just one factor to consider when assessing whether pesticides pose a risk to salmon populations. An additional factor is the relative sensitivity of salmon and their prey to various pesticides. For some insecticides, a concentration that kills invertebrates may also cause sublethal effects in fish (i.e., reducing AChE activity); however, for others, concentrations that are lethal for invertebrates may have few if any sublethal effects on fish (e.g., Table 1). Therefore, insecticides found in surface waters may affect a salmon’s ability to feed but may also kill much of their prey [1], [15], [16]. Juvenile salmon feed opportunistically on invertebrates drifting in the water column [12], so reductions in these invertebrates may affect salmon growth and survival as much or more than a reduced ability to feed [17], [18]. Because the sensitivities of salmon and their prey differ (e.g., Table 1), each insecticide may have a different potential to affect salmon via reducing their capacity to feed or through the reduction of prey itself.

**Table 1:** Effects concentrations (μg/L) and slopes for salmon AChE activity, and prey abundance dose-response curves for several organophosphate (OP) and carbamate (CB) insecticides.

Insecticide	Class	Salmon AChE activity		Prey abundance		AChE EC_50_ Prey EC_50_
		EC_50_	slope	EC_50_	slope	
Chlorpyrifos	OP	2.0	1.50	2.30	1.8	0.9
Diazinon	OP	145.0	0.79	1.38	1.8	105.1
Carbaryl	CB	145.8	0.81	4.33	5.5	33.7

The ratio of the AChE EC_50_ to prey EC_50_ illustrates the relative sensitivities of the salmon AChE activity and their prey abundances to the insecticide. Salmon AChE values are from Laetz et al. [29]. Details of how prey abundance values were derived are given in Supporting Information S1.

Considering how insecticides affect the dynamics of aquatic invertebrate communities may also be relevant for assessing impacts on salmon [19]. Insecticides can cause catastrophic invertebrate drift [20], with dead or moribund invertebrates leaving the benthos and flowing downstream in the water column at rates more than 1000 times greater than normal levels (e.g., [21], [22]). Juvenile salmon may feed on this temporary “spike”, or excess in prey [23], but prey may be depleted for many months following such events [20], [21], [23], [24]. The amount of prey available over time will depend on how vulnerable the invertebrate community is (or how low to some “*prey floor*” it is driven), and how quickly the invertebrate community can rebound (*prey recovery rate*). Because it is unlikely that there is a single prey recovery rate or a single value that reflects the proportion of prey that could persist following an extreme exposure, a range of these values should be evaluated when considering the ways fluctuations in prey affect salmon feeding and growth.

The properties of an insecticide that contribute to its toxicity may also influence the likelihood that individual salmon and their prey are exposed. The environmental persistence of an insecticide, as well as the dynamics of the targeted pest, may influence how frequently it is applied. Repeated applications may be needed for controlling some pests, while a highly persistent insecticide may be applied only once because it remains toxic for several months. Consequently, there may be sustained or repeated exposures depending on how often pesticides are applied throughout a watershed. In addition, the application technique (e.g., applied aerially vs. on the ground), as well as weather (e.g., the frequency and intensity of rain, wind), will influence how likely insecticides contaminate aquatic habitats. Therefore, when evaluating whether an insecticide could harm salmon and their prey, researchers must consider the timing, frequency and duration of a likely exposure.

Although data gaps and uncertainties remain, there is increasing recognition that a more comprehensive approach is needed for evaluating the potential effects of pesticides on non-target communities [16], [25]–[28]. Notably, the National Research Council [28] recently recommended including potential population-level impacts of any sublethal (e.g., impaired behavior) and indirect (e.g., reduced prey) effects of pesticide exposures when assessing impacts on threatened and endangered species, including Pacific salmon. This is challenging, as it requires quantifying the interactions among salmon and their prey as they are altered by the type, timing, frequency, duration and intensity of pesticide applications. When evaluating the population-level impacts of all of these factors, an ecological modeling approach can provide valuable insight.

In evaluations of pesticides and their potential effects on Pacific salmon listed as threatened or endangered under the US Endangered Species Act (ESA) [17], [18], the National Marine Fisheries Service (NMFS) has laid the groundwork for modeling sublethal and indirect impacts of these chemicals on salmon populations. Here, using and expanding upon these models, we compare the relative importance of direct and indirect effects of several insecticides on salmon populations, and explore how population-level effects are influenced by the dynamics of salmon prey. To do this, we incorporate prey dynamics into a model that examines the population-level impacts of direct effects of pesticides [11]. Three parameters describing prey community dynamics following an exposure - a one-day spike, the prey floor or lowest level to which prey abundance can be reduced, and the recovery rate - were incorporated in the model. Here we describe how those parameters affect overall salmon population growth rates. In addition, we examine how the frequency, duration and timing of insecticide exposure interact with prey dynamics in their effect on salmon population growth rates.

## Methods

We modified a model previously developed by Baldwin et al. [11] to assess the potential effects of AChE-inhibiting pesticides (n-methyl carbamate and organophosphate insecticides) on Pacific salmon at the individual and population scales. At the individual scale, the modified model links chemical exposure to reductions in feeding behavior and prey abundance, food intake and, by extension, the juvenile somatic growth of ocean-type Chinook salmon (*O. tshawytscha*). At the population scale, the model utilizes the relationship between subyearling size at ocean migration and subsequent size-dependent mortality, to evaluate corresponding consequences for population growth rate across multiple generations. We modeled varying pesticide exposures in freshwater habitats for juvenile salmon to estimate how changes in individual somatic growth may influence population-scale abundance, as indicated by a reduction in the intrinsic rate of increase, or λ. Modeled exposure concentrations spanned the known ranges of toxicological sensitivities for salmon and their prey. The model was constructed using MATLAB 7.9.0 (R2009b) (The MathWorks, Inc. Natick, MA).

### Individual-based modeling

The organismal portion of the model tracked the somatic growth of individual salmon to assess how a pesticide exposure may act through effects on salmon feeding and on prey abundance. For the direct effects on the salmon feeding we quantified the physiological pathway between AChE activity and the somatic growth of salmon fingerlings based upon a series of empirical relationships between pesticide exposure, AChE inhibition, feeding behavior, food uptake, and somatic growth rate. The relationship between exposure and AChE inhibition includes the EC_50_ (the concentration that produces 50% AChE inhibition) and the slope of the exposure response curve. For each pesticide, values for the EC_50_ and slope were from Laetz et al. [29]. Further descriptions of the relationships linking the direct effects of AChE inhibition on salmon feeding and growth can be found in Baldwin et al. [11].

For the indirect effects on salmon via their prey, we used empirical data to develop a relationship between pesticide exposure and prey abundance (i.e. the ration of food available for individual juvenile salmon). This relationship includes the EC_50_ (the concentration that would reduce available prey by 50%) and the slope describing the sensitivity of prey to a range of pesticide exposures. Invertebrate toxicity data were obtained from the U.S. Environmental Protection Agency’s Ecotox database (http://cfpub.epa.gov/ecotox/) and from replicated mesocosm experiments (e.g., [30]), and were used to generate a single, representative EC_50_ and slope for each pesticide (Table 1). Only toxicity data from studies on taxa known to be salmon prey, or ecological or physiological surrogates, were included in calculating the prey community EC_50_s. Details can be found in the Supporting Information S1 and Figure S1.

**Figure 1:**
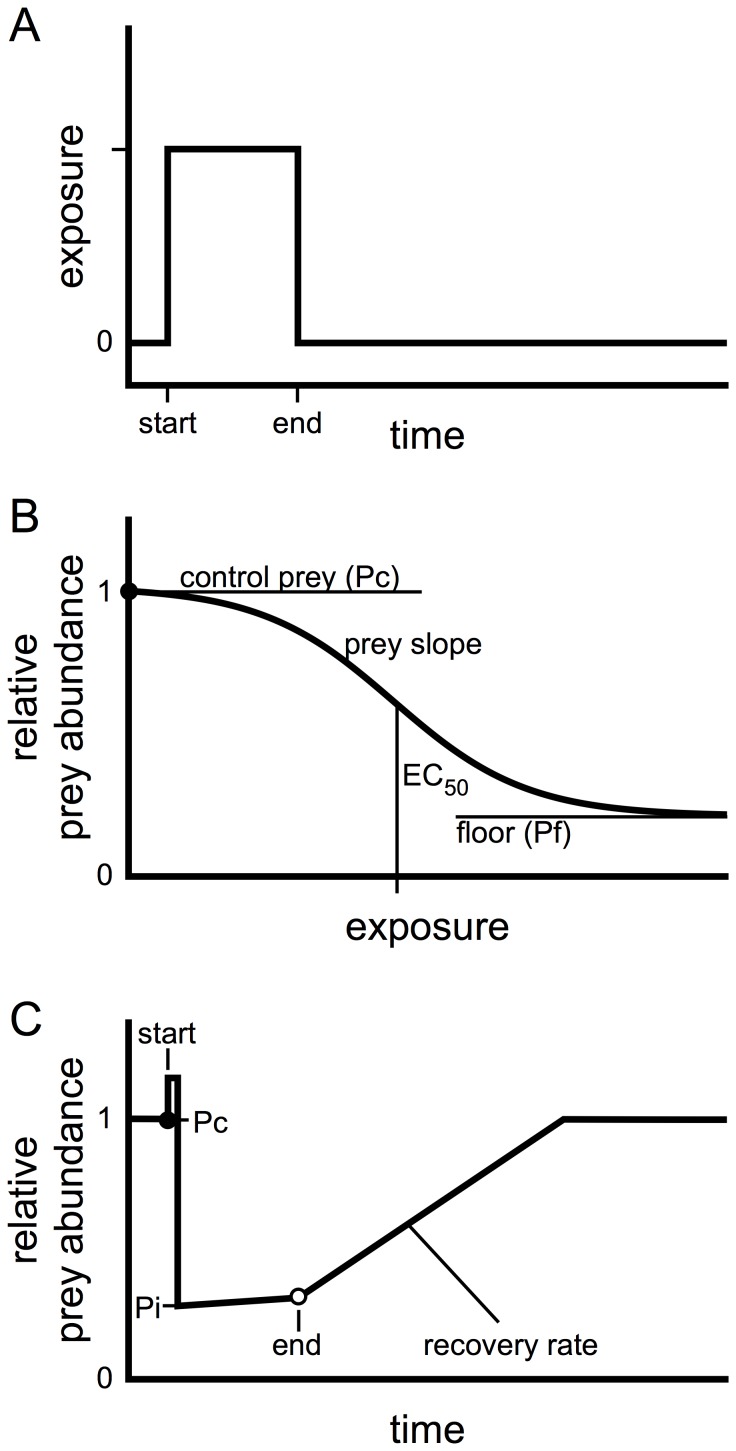
Relationships used to link anticholinesterase exposure to the abundance of prey. A) Step pulse of exposure to an anticholinesterase pesticide. B) Sigmoidal relationship between exposure concentration and relative prey abundance defined by control abundance (Pc), sigmoid slope (prey slope), prey EC_50_, and a minimum abundance (prey floor, Pf). C) Time course of prey abundance in response to a step exposure. Pc denotes the control prey abundance before the exposure. Pi denotes the reduced prey abundance at the start of the exposure.

**Figure 2:**
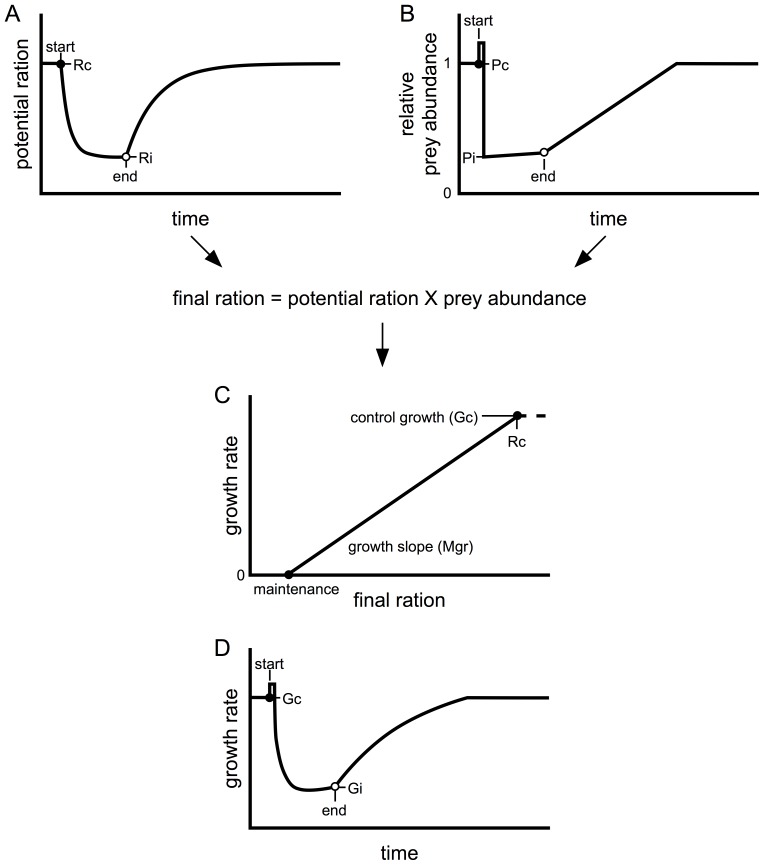
Relationships linking anticholinesterase exposure to individual ration and growth rate. A&B) Relationships describing the time course of the effects of exposure on the organisms ability to capture food (A, potential ration) and the availability of food (B, relative prey abundance). C) Linear model linking final ration (potential ration times relative prey abundance) to growth rate using a line passing through the control condition with a slope denoted by Mgr. D) Time course for effect of exposure on individual growth rate produced by combining A, B, & C. See text for details. Closed circles represent the control condition just prior to exposure, and open circles (e.g. Ai) represent the exposed (inhibited) condition at the end of the exposure.

The relationships in the organismal portion of the model utilize steady state sigmoidal dose-response curves to link pesticide exposure with the effects on salmon AChE activity (see [11]) and relative prey abundance. The sigmoidal curves were defined using specific EC_50_s and slopes (Table 1, Figures 1 and 2). Pesticide exposures in the organismal portion of the model were defined by pulses of various lengths (i.e. number of days) and timing (i.e. day exposure begins) with the pesticide concentration during a single pulse remaining constant (Figure 1A). The relative prey abundance concentration response curve was derived from the prey EC_50_ and slope, and bound between the control abundance and a defined prey floor (Figure 1B). The prey floor is the portion of the prey community that remains regardless of a pesticide exposure. This accounts for a small but constant input of unaffected terrestrial insects into salmon habitats as well as tolerant (pesticide resistant) aquatic invertebrates. For each scenario the exposure concentration was calculated for each time point (Figure 1A) and, using the exposure to relative prey abundance relationship (Figure 1B), the time course for relative prey abundance was determined (Figures 1C and 2B). The time course for relative prey abundance and related available ration also incorporated a one-day spike in prey drift, with the magnitude of the drift depending on both the toxic potency of the pesticide and the sensitivity of the available prey community. The transient spike was followed by a sustained drop in prey abundance and then a gradual recovery (Figure 1C). The size of the prey spike was estimated as a 20-fold increase over the standing prey abundance on the day prior to pesticide exposure, minus the prey floor. Prey recovery was assumed to be constant, reflecting a constant influx of invertebrates from connected habitats. During an exposure any new invertebrates recruited into habitats were subject to the toxicity and the rate of prey recovery was adjusted to capture the additional losses. Prey recovery continued until control (pre-exposure) drift rates were reached or another exposure occurred. The parameter values defining control baseline conditions and exposure scenarios are listed in Tables 2 and 3, respectively.

**Table 2:** List of values used for control parameters to model organismal growth and the model sensitivity to changes in the parameter.

Parameter	Value^1^	Error^2^	Sensitivity^3^
AChE activity (Ac)	1.0^4^^,^ 5	0.06^5^	–0.167
feeding (Fc)	1.0^4^^,^ 5	0.05^5^	0.088
ration (Rc)	5% weight/day^6^	0.05^7^	–0.547
feeding vs. activity slope (Mfa)	1.0^5^	0.1^5^	–0.047
ration vs. feeding slope (Mrf)	5 (Rc/Fc)	-	-
growth vs. ration slope (Mgr)	0.35^6^	0.02^6^	–0.547
growth vs. activity slope (Mga)	1.75 (Mfa*Mrf*Mgr)	-	-
initial weight	1 gram^8^	0.1^8^	1.00
control prey drift	1.0^4^	0.05^11^	0.116
AChE impact time-to-effect (t_1/2_)	0.5 day^9^	n/a	0.005
AChE time-to-recovery (t_1/2_)	0.25 days, 30 days^10^	n/a	–0.0001
prey floor	0.05, 0.20, 0.50^11^	n/a	0.178
prey recovery rate	0.005, 0.01, 0.05^12^	n/a	0.323
somatic growth rate (Gc)	1.3^13^	0.06^6^	2.531

1 mean value of a normal distribution used in the model or constant value when no corresponding error is listed.

2 standard deviation of the normal distribution used in the model.

3 mean sensitivity when baseline parameter is changed over range of 0.5 to 2-fold, S =  (change in final/baseline weight)/(change in parameter/baseline parameter).

4 other values relative to control.

5 derived from [4].

6 derived from [31].

7 data from Brett et al. [31] has no variability (ration was the independent variable) so a variability of 1% was selected to introduce some variability.

8 consistent with field-collected data for juvenile Chinook [64].

9 estimated from [9].

10 0.25 days consistent with [8]; 30 days from [65].

11 range estimated from [21], [41], [43].

12 range estimated from [24], [43], [44].

13 derived from [31] and adapted for ocean-type Chinook.

**Table 3:** Model scenarios and the questions they aim to address.

Question	approach	class/compound	Exposure parameters				Salmon AChE parameters				Prey parameters		
			number of pulses	pulse duration, days	pulse start, day(s)	EC_50_, μg l^−1^	slope	recovery rate, days	EC_50,_ μg l^−1^	slope	recovery rate, % day^−1^	spike	floor
**What is the relative importance of direct vs. indirect impacts on salmon populations?**	Compare effects of 4 insecticides on salmon alone vs. salmon and their prey	chlorpyrifos	1	4	30	**2**	**1.5**	**30**	**2.3**	**1.8**	1	yes	0.2
		diazinon	1	4	30	**145.0**	**0.79**	**30**	**1.38**	**1.8**	1	yes	0.2
		carbaryl	1	4	30	**145.8**	**0.95**	**0.25**	**4.33**	**5.5**	1	yes	0.2
		hypothetical carbamate	1	4	30	**2**	**1.5**	**0.25**	**2.3**	**1.8**	1	yes	0.2
**How do the dynamics of response of the prey community affect salmon populations?**	Vary prey recovery rate	carbaryl	1	4	30	145.8	0.95	0.25	4.33	5.5	**0.5; 1; 5**	yes	0.2
	Vary prey resistance level or floor	carbaryl	1	4	30	145.8	0.95	0.25	4.33	5.5	1	yes	**0.05; 0.2; 0.5**
	Compare with and without prey spike	carbaryl	1	4	30	145.8	0.95	0.25	4.33	5.5	1	**yes & no**	0.2
**How do the frequency, duration & timing of exposures affect salmon populations?**	Vary frequency, duration and timing of exposures	carbaryl	**1, 2, 4, & 8**	**4, 8, 16, & 32**	**See ** **Fig. 7**	145.8	0.95	0.25	4.33	5.5	**0.5; 1; 5**	yes	**0.05; 0.2; 0.5**

Parameters that were changed in each scenario are in bold.

The direct physiological effect of an exposure will determine a fish’s ability to feed (Figure 2A, [11]). The final ration consumed by a fish is dependent on both how much food it is capable of eating (i.e. potential ration) and on how much food is available (i.e. relative prey abundance).The final ration available each day was the product of potential ration and the relative prey abundance (outputs of Figures 2A and 2B). The amount of prey (Figure 2B) that could be consumed during a prey spike was capped at a maximum of 1.5 times the control drift since a fish’s maximum feeding capacity limits the amount of excess food it can exploit. The size change for individual fish each day was calculated from the final ration and somatic growth rate (Figure 2C, [31]). An example of the individual somatic growth rate over time is provided in Figure 2D.

The organismal growth model was run for 1000 individual fish, with initial weight selected from a normal distribution with a mean of 1.0 g and standard deviation of 0.1 g. This weight was representative of juvenile Chinook salmon in the early spring, before the seasonal application of insecticides. For each day modeled, the somatic growth rate and fish weight were calculated using parameter values selected from their normal distributions (Table 2). Normal distributions appropriately represented the literature and experimental data and were extended to parameters for which values were estimated. This was repeated each day for the 140 days of the subyearling freshwater growth period across 1000 fish. Further details can be found in Baldwin et al. [11]. The organismal model run produces a mean weight and standard deviation for the subyearling salmon, which is the input for the size-dependent first year survival function of the population model. A sensitivity analysis was run to determine the influence of the organismal model input parameter values on the final somatic size by altering each baseline value by 0.5 to 2 fold (Table 2).

### Population-scale modeling

The subyearling weight distribution was used to calculate size-dependent first-year survival for an ocean-type Chinook life-history matrix population model [11]. A brief description is provided here and details can be found in Baldwin et al. [11]. The first-year survival element of the transition matrix incorporates a size-dependent survival rate for a three-month interval, which is the last three months of their first year. This represents the period the subyearling smolts spend in estuarine and nearshore habitats. The weight distributions (based on the calculated mean and standard deviation) from the organismal model were converted to length distributions by applying condition factor calculations from length and weight relationships collected for subyearling ocean-type Chinook [32].

The relationship between an individual salmon’s length and the rate of survival during migration and estuary residence was adapted from Zabel and Achord [14] to match the survival rate for the unexposed Chinook salmon population [33], [34]. The relationship is based on the length of a subyearling salmon relative to the mean length of competing subyearling salmon of the same stock, (Δlength), and relates that relative difference to size-dependent survival. A size-dependent survival probability for each fish was generated by randomly selecting length values from the normal distribution calculated from the organismal growth model. This process was replicated for 1000 modeled Chinook salmon for each exposure and a corresponding unexposed cohort to yield mean size-dependent survival rates which were inserted into the first-year survival calculation for the two groups.

To determine population-level responses, an age-structured life history model was constructed for ocean-type Chinook salmon. The model assumed a maximum female age of 5 with reproductive maturity at ages 3, 4 or 5 [11]. Transition values were determined from the literature on survival and reproductive characteristics from several ocean-type Chinook populations in the Columbia River system [33], [35]–[39]. The spawner sex ratio was approximately 1:1. Age-based fecundity, number of eggs (standard deviation), of 4511 (65), 5184 (89), and 5812 (102) for 3,4, and 5-year olds was calculated using length data from Howell et al. [33], and the length-fecundity relationships from Healey and Heard [35]. Control survival and reproduction matrix values for the model are listed in Table 4.

**Table 4:** Matrix transition element and sensitivity and elasticity values.

Transition Element	Ocean-type Chinook Salmon		
	Value^1^	Sensitivity	Elasticity
S1	0.0056	57.13	0.292
S2	0.48	0.670	0.292
S3	0.246	0.476	0.106
S4	0.136	0.136	0.0168
R3	313.8	0.0006	0.186
R4	677.1	0.000146	0.0896
R5	1028	1.80E-05	0.0168

1 Values calculated from data in [32], [33], [35]–[39].

Analysis of the transition matrix, A, explored the intrinsic population growth rate as a function of the survival and reproductive rates [40]. The intrinsic population growth rate, i.e. lambda (λ), equals the dominant eigenvalue of A and was calculated using matrix analysis software noted above. Variability was integrated by repeating the calculation of λ 2000 times, selecting the values in the transition matrix from their normal distribution defined by the mean standard deviation for each calculation. Normal distributions appropriately represented the literature data. The population model output consists of the percent change in intrinsic population growth rate (%Δλ; mean and standard deviation) of the pesticide-exposed population from the control population. Change in λ is a population parameter often used by the National Marine Fisheries Service as well as other federal, state and local agencies in evaluating population productivity, status, and viability. Sensitivity and elasticity analyses were conducted on the control transition matrix as reported previously [11]. The influence of each matrix element, a_ij_, on λ was assessed by calculating the sensitivity values for A. The sensitivity of matrix element *a_ij_* equals the rate of change in λ with respect to *a_ij_*, defined by δλ/δ*a_ij_*. The elasticity of matrix element *a_ij_* is defined as the proportional change in λ relative to the proportional change in *a_ij_*, and equals (*a_ij_*/λ) times the sensitivity of *a_ij_.* Higher sensitivity and elasticity values indicate greater influence on λ [39].

The populations were assumed to be density independent and closed (i.e. no net migration in or out of the population in the form of straying). Consistent data were lacking to establish any type of density dependent relationship. Since certain forms of density dependence can influence population dynamics through compensation and other demographic processes, using density independence minimizes the likelihood of a Type 2 error arising from inaccurate assumptions of density-dependence. No stochastic impacts are included beyond natural variability in all parameters. This was represented by selecting all parameter values from a normal distribution about their mean for each model step. Non-pesticide influences on population dynamics, including ocean conditions, fishing pressure, and marine food availability were assumed constant and density independent. Each individual fish experienced a pesticide exposure scenario only as a subyearling (during its first spring) and the exposure scenario was assumed to occur annually to all subyearlings in the population.

### Scenarios

Using the model, we ran specific scenarios (Table 3) to assess 1) the relative importance of direct vs. food web-mediated pesticide impacts on salmon population growth rates, 2) the influence of prey community sensitivity and response dynamics on salmon population growth rates, and 3) how the frequency, duration and timing of pesticide exposures affect Chinook salmon population growth rates. These scenarios and their parameters are listed in Table 3.

#### 1 The relative importance of direct vs. food web-mediated pesticide impacts on salmon population growth rates

For the first set of scenarios, we considered several insecticides and, for each, compared model runs of either only direct effects or both direct and indirect effects (Table 3). Four pesticides were selected to illustrate how two factors associated with the pesticide – the salmon AChE activity recovery time and the relative toxicity between salmon and their prey - influence the relative importance of the direct and indirect effects of exposure. First, we considered two classes of insecticides, organophosphates and carbamates, which differ in how long it takes salmon AChE activity to recover following exposure (recovery half-lives of 30 days and 6 hours, respectively, Table 3). Second, we used insecticides that differ in their relative toxicity to salmon and their prey. Chlorpyrifos, an organophosphate, has an EC_50_ for brain AChE inhibition in salmon that is similar to the EC_50_ for invertebrate prey mortality (Table 1). By contrast, diazinon (also an organophosphate) is more toxic to invertebrates than to salmon (Table 1). Carbaryl, a carbamate, is similar to diazinon in that it is more toxic to invertebrate prey than salmon (Table 1), but unlike diazinon, salmon can recover rapidly from a sublethal carbaryl exposure (Table 3). In order to make the comparison between the classes of pesticides while controlling for the relative toxicities, we considered a hypothetical carbamate that would be similar to chlorpyrifos in all ways except salmon AChE activity recovery time (Table 3). By comparing this hypothetical carbamate along with the three actual insecticides, we assessed how salmon AChE activity recovery rates and the relative toxicity of insecticides influence the importance of direct vs. indirect effects.

#### 2. The influence of prey community sensitivity and response dynamics on salmon population growth rates

To address this second point, we developed scenarios using the carbamate carbaryl to examine how specific prey dynamics affect the impact of exposure on salmon population growth rates, since the impacts of this pesticide are primarily due to effects on prey. We ran the model using a range of ecologically relevant values for the prey community’s recovery and resistance (Table 3). The rate at which invertebrate communities recover following pesticide exposures varies widely. In some systems, invertebrate densities and biomass return to background or reference levels within weeks of exposure, while in others it can take months to years to recover [24], [41]–[45]. Invertebrate community recovery rates of around 1% per day have been reported frequently [41], [43], and therefore, we used that as the prey recovery rate for most of the scenarios. We also used slower and faster rates in additional scenarios to explore how different prey recovery rates affect salmon population growth rates (Table 3; [24], [46]).

To explore how the resistance or tolerance of the prey community to insecticide toxicity translates to salmon population growth rates, we set the prey floor (the lowest possible ration available following exposures) at 0.05, 0.20 or 0.50. These values indicate under extreme exposures (i.e., those exceeding the invertebrate EC_50_), the available ration could be reduced by as much as, but not more than, 95%, 80%, or 50%, respectively. While no studies specify prey floors per se, field studies quantifying the impacts of highly toxic pesticide exposures on invertebrates indicate this range is realistic [21], [41], [43], [47]–[49]. Because some of the most carefully executed experiments report <10% to 25% of the community may persist after exposures to pesticides [21], [41], [50], we set the prey floor at 0.20 for most scenarios (Table 3). In addition, to assess the importance of a one-day spike in prey to salmon population growth, we ran scenarios with and without the spike (Table 3).

#### 3. The influence of the frequency, duration and timing of pesticide exposures on salmon population growth rates

To address this, we examined the output of scenarios that varied in the duration, frequency and timing of carbaryl exposures (Table 3). To examine the effect of changing the exposure duration, the model was run using a constant exposure lasting 4, 8, 16, or 32 days. To assess the effect of exposure frequency, a 4-d exposure was repeated either 1, 2, 4, or 8 times. Finally, to assess the impact of exposure timing, different intervals between multiple exposures were used. All combinations were run and those representing the full range of output are presented in the results.

## Results

### Sensitivity Analyses

A sensitivity analysis conducted on the organismal model revealed that changes in the control somatic growth rate had the greatest influence on the final weights of juvenile salmon (Table 2). While the somatic growth value was experimentally derived for sockeye salmon [31], this value was adapted for ocean-type Chinook salmon and is within the variability reported in the literature for other salmon (reviewed in [51]). Other parameters related to the daily growth rate calculation, including the growth to ration slope (Mgr) and control ration, produced strong sensitivity values. Initial weight, prey recovery rate and prey floor also strongly influenced final weight values (Table 2). Large changes (0.5 to 2 times) in other key parameters produced proportionate changes in final weight. The sensitivity analysis of the control population matrix predicted the greatest changes in population growth rate result from changes in first-year survival (Table 4). The standard deviation for the control population matrix was on average 10.2 and for the exposed population matrices ranged from 6.8 to 11.6. The elasticity values for the transition matrix also corresponded with the driving influence of first-year survival.

### The relative importance of direct vs. food web-mediated pesticide impacts on salmon population growth rates

**Figure 3:**
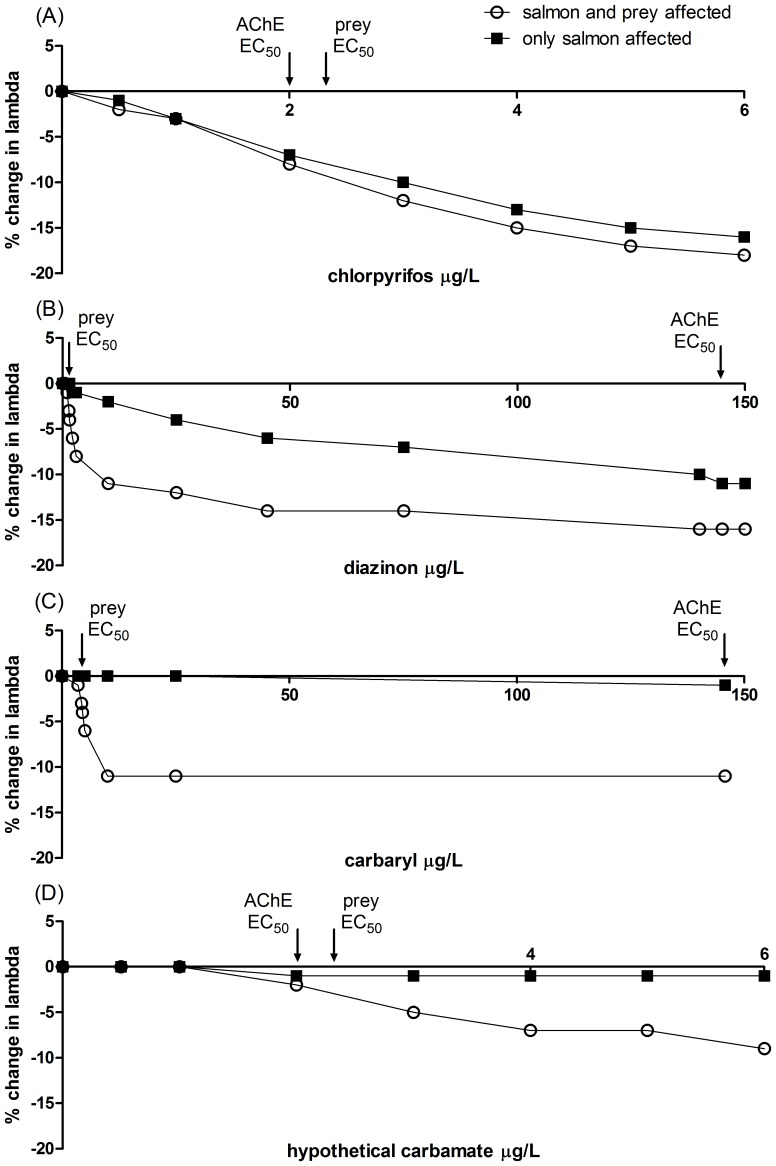
Change in salmon population growth rates due to direct and indirect effects of pesticides. Mean percent change in population growth rates (%Δλ) between unexposed ocean-type Chinook salmon and those exposed to a single, 4-day exposure per generation of chlorpyrifos (A), diazinon (B), carbaryl (C), and a hypothetical carbamate (D) as generated by the model. Scenarios for each insecticide include one with only the direct effects on salmon and another with effects on both salmon and their prey.

The relative importance of direct effects of pesticide exposure on salmon feeding behavior versus indirect effects via reductions in their prey depends not only on how quickly salmon recover from an exposure but also on how sensitive salmon are compared to their prey. If salmon are slow to recover following exposure, and their AChE EC_50_ is similar to the EC_50_ of their prey (e.g., chlorpyrifos, Figure 3A), almost all of the impact on salmon population growth rates (λ) results from the direct effect on fish. Reductions in prey have little additional effect on the percent change in salmon population growth rates (%Δλ), regardless of the exposure concentration (Figure 3A). When the relative toxicity of the organophosphate is much greater for prey than for salmon (e.g., diazinon, Table 1 and Figure 3B), the direct effect on fish is still important, however, reductions in prey are also important. For diazinon, reductions in prey contribute much more to the overall %Δλ, particularly at lower concentrations (e.g. just above the prey EC_50_ but well below the salmon EC_50_). Therefore, for insecticides like organophosphates that can have sublethal but long-acting effects on individual salmon, the difference in the relative toxicity of the insecticide to the salmon compared to their prey determines how important direct effects on salmon are compared to the reductions in their prey.

In contrast, when salmon are able to recover quickly (e.g., the insecticide is a carbamate instead of an organophosphate), reductions in salmon population growth rates are due almost entirely to reductions in prey and not to sublethal toxicity to the fish (Figure 3C and 3D). The example of the hypothetical carbamate (Figure 3D) illustrates that this is the case regardless of the relative toxicity of the carbamate on salmon versus their prey. For instance, even if we assume the AChE EC_50_ and prey EC_50_ are similar for this hypothetical carbamate, the effect of reduced prey produces a greater %Δλ (Figure 3D). Thus for insecticides that can have sublethal but brief effects on salmon, reductions in prey are more important in reducing salmon population growth rates than direct effects on feeding behavior, regardless of how sensitive salmon are compared to the prey.

### The influence of prey community sensitivity and response dynamics on salmon population growth rates

**Figure 4:**
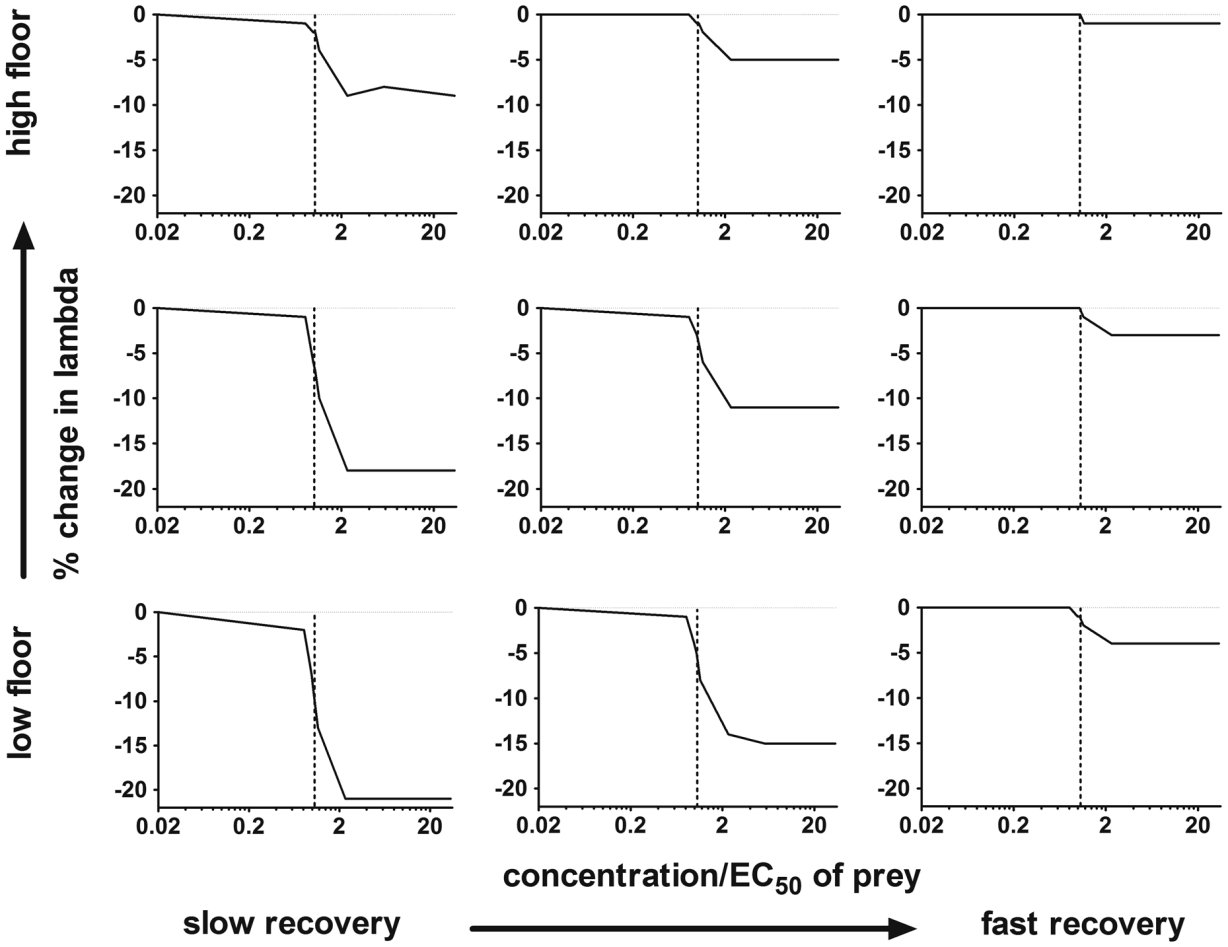
Effect of prey recovery rate and prey floor on salmon population growth rates. Mean percent change in lambda (on the y-axes) between modeled populations of unexposed ocean-type Chinook salmon and those exposed to a single, 4-day exposure per generation of a carbamate insecticide (e.g., carbaryl). The nine scenarios vary in the prey recovery and prey floor parameters (Table 3). Prey recovery rates were 0.5%, 1% and 5% per day, or slow, intermediate and fast, respectively. Prey floors were 0.05, 0.20 and 0.50, or low, intermediate and high, respectively. The vertical dotted line in each panel marks where the exposure concentration equals the EC_50_ of the prey (4.33 μg/L for carbaryl).

For single, 4-day pulses of an insecticide like carbaryl (in which effects on prey dominate), the magnitude of the %Δλ depends on both the prey recovery and prey floor (Figure 4). For the scenario with the intermediate values for prey recovery rate and the prey floor, the mean reduction in salmon population growth rates was –11% at the highest concentration considered (145.8 μg/L carbaryl, which is the EC_50_ of the salmon AChE activity and 33.7 times the EC_50_ of the prey). At this same concentration, a scenario with a fast recovery and a high prey floor resulted in %Δλ of only –1%. At the other end of the spectrum, a scenario assuming slow prey recovery and a low prey floor resulted in %Δλ of –21% at the highest concentration considered. The magnitude was lower at more environmentally realistic concentrations, but the range in %Δλs indicated the prey recovery rate and the prey floor remain important parameters in understanding potential effects on salmon population growth rates. For instance after a single, 4-day pulse at a concentration equivalent of 1.15 times the prey EC_50_ (e.g. 5 μg/L carbaryl), model outputs ranged in mean %Δλ from –1% to –13% (Figure 4) depending on the prey recovery rate and the prey floor.

Although the range of responses across these scenarios indicates both the prey recovery rate and the prey floor affect the %Δλs, the prey recovery rate may be particularly important. The range of %Δλs was especially wide across the prey recovery rates considered (0.5, 1 and 5%/day; Figure 4). The slow prey recovery rate resulted in declines in salmon population growth rates (%Δλs of –9 when the exposure was 2 times the prey EC_50_) even when as much as 50% of the prey were unaffected (i.e. prey floor was 0.5, Figure 4). In contrast, when the prey floor was at the lowest level considered (0.05) such that 95% of the available prey could be eliminated following an exposure, salmon population growth rates were only moderately reduced if the prey recovery rate was fast at 5%/day (%Δλ of –4 when the exposure was 2 times the prey EC_50_).

**Figure 5:**
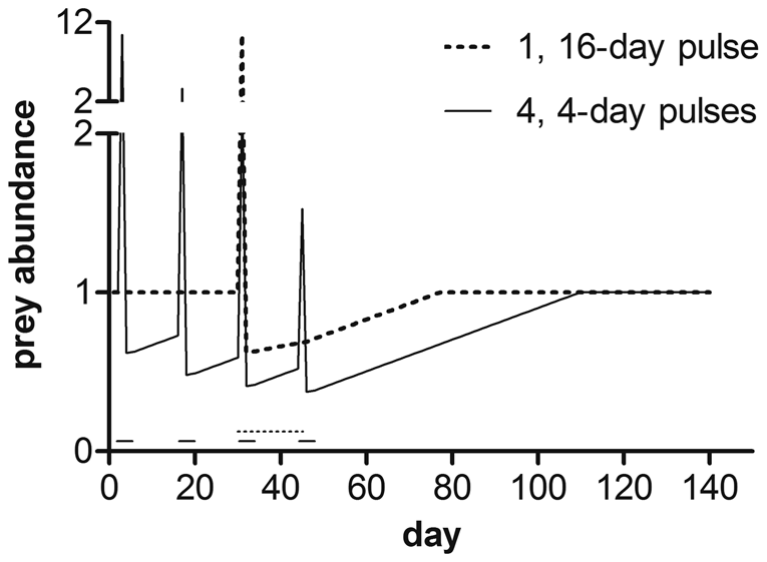
Effect of sustained or pulsed exposure on prey abundance. Prey available per day (relative to control) for juvenile ocean-type Chinook salmon in scenarios in which fish and prey were exposed to carbaryl at the prey EC_50_ (4.33 μg/L) for either 1, 16-day pulse starting at day 30 (dates noted by dotted line), or 4, 4-day pulses starting on days 2, 30, 58 and 86 (noted by solid lines). Prey floor and prey recovery rates were intermediate (0.2 and 1% per day, respectively) for both scenarios. Model output indicated %Δλs for 1, 16-day and 4, 4-day exposure scenarios were –3 (9.8) and –12 (8.9), respectively.

The inclusion of the prey spike caused interesting fluctuations in prey abundance immediately following exposure(s) (Figure 5), but this short-term effect had almost no lasting effect on salmon population growth rates. Two scenarios were run with and without spikes (a single 4-d pulse and 4, 4-d pulses, each with prey recovery  =  1% and prey floor  =  0.20) at seven concentrations (ranging from 0.8 to 33.7 times the prey EC_50_, or 3.5 to 145.8 μg/L carbaryl). For the single pulse exposure, the spike ameliorated the impact but only minimally and only at the highest concentration (e.g., %Δλs  =  –11 and –12 at 145.8 μg/L carbaryl with and without spike, respectively). The inclusion of prey spikes following each of the 4, 4-day pulses in the second scenario also had a minimal but more frequent effect; the inclusion of spikes lessened the impact on λ slightly across all but one of the concentrations (difference between %Δλs  =  1). The apparent effect of the spike parameter was minimal compared to the prey recovery rate and prey floor parameters, largely because the spike only influences ration for 1 day per exposure pulse, out of the subyearling salmon’s 140-day growth period.

### The influence of the frequency, duration and timing of pesticide exposures on salmon population growth rates

**Figure 6:**
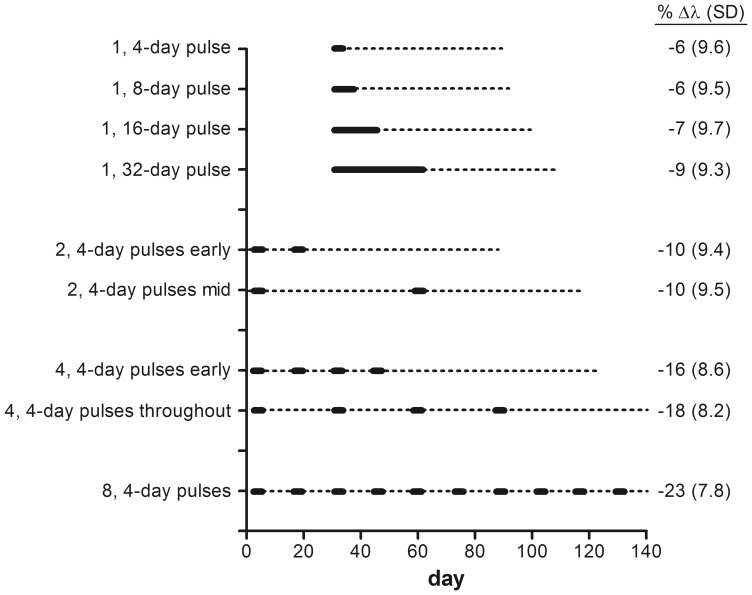
Effect of exposure timing and duration on salmon population growth rates and prey abundance. Timing and duration of exposures and results from 9 scenarios run with 1.15 times prey EC_50_ (5.0 μg/L) of carbaryl. Dark solid lines indicate the timing and duration of exposure(s). Dotted lines indicate period after each exposure in which prey abundance was reduced (ration <1). Values listed after scenarios are the %Δλ (SD) for ocean-type Chinook salmon, for exposures of 5.0 μg/L. Prey recovery rate was 1% per day and the prey floor was 0.20.

**Figure 7:**
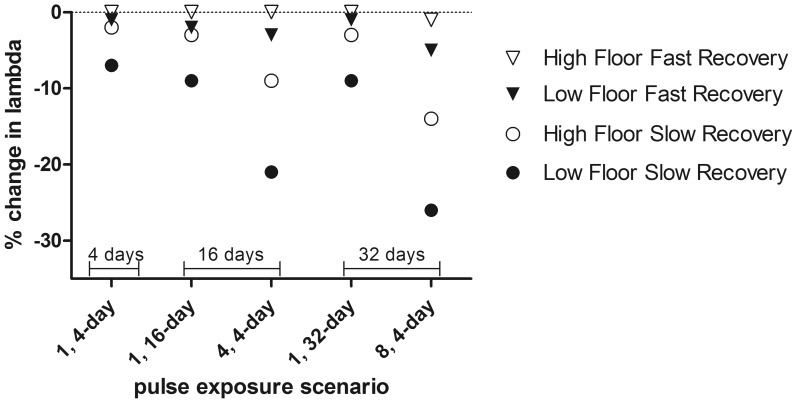
Effect of pulses, prey recovery and prey floor on salmon population growth rates. Mean percent change in lambda of ocean-type Chinook salmon following various exposures to carbaryl. All exposures were at the prey EC_50_ (4.33 μg/L), but the duration and frequency of the exposures varied as well as the floor and recovery rates for the prey. Low and high prey floors were 0.05 and 0.5; slow and fast prey recovery rates were 0.5% or 5% per day. The total days of exposure are noted with brackets.

Varying the duration, frequency and timing of the pesticide exposures affected the modeled impacts on salmon population growth rates (Figures 6 and 7). Longer or more frequent exposures resulted in greater reductions in prey abundance and subsequent reductions in salmon population growth rates. For example, increasing the duration of a single pulse increased the effect on populations; exposing populations for 32 days instead of 4 days resulted in %Δλ –9 compared to %Δλ –6, respectively (at 5 μg/L carbaryl, with prey recovery  =  1% and prey floor  =  0.20, starting at day 30, Figure 6). Figures 6 and 7 illustrate how short frequent exposures (e.g., 4, 4-d pulses; 16 total days of exposure) had a greater effect on salmon population growth rates than long continuous exposures (e.g., 1, 32-d pulse; 32 total days of exposure), even when the actual duration of the exposure was longer in the latter. This occurs due to the additional time the prey community takes to recover from multiple exposures (Figure 6). For example, prey abundance was reduced to a greater extent for a longer portion of the growing period after four, 4-day pulses than after a single, 16-day pulse (Figure 5). The importance of prey recovery rate is especially clear when comparing salmon population-level impacts from single, long versus multiple, short exposure pulses (Figure 7).

We found the number of repeated exposures influenced salmon population growth rates more than the timing of the exposures. Figure 6 illustrates how the timing of exposures affected the length of time prey were reduced throughout the season. Although not shown, the model output also indicated prey abundance on any given day varied depending on the timing of exposures. While this may have implications for designing monitoring studies to assess prey abundance, for most scenarios the shift in timing of prey abundance had little overall effect on salmon population growth rates (Figure 6).

## Discussion

Our analyses show current use pesticides at modeled exposure concentrations may affect salmon population growth rates both directly, though changing their feeding behavior, and indirectly, by reducing their prey. For some insecticides, reductions in prey alone are sufficient to reduce salmon population growth rates. The potential impact depends on the frequency and duration of the exposure concentration, as well as the dynamics of the prey community. Prey communities that are relatively tolerant and can recover quickly may be able to sustain juvenile salmon, while less resistant invertebrate communities that cannot rebound quickly may not be productive enough to support robust salmon populations.

### The class of insecticide and its relative toxicity to salmon vs. prey are key factors affecting the relative importance of direct vs. indirect effects on salmon population growth rates

The differences in salmon population growth rates following modeled exposures to various insecticides illustrate the critical need for understanding the diversity of possible toxicological effects that pesticides may have on a species and its habitat. These examples demonstrate how different classes of insecticides may have very different effects on various components of a food web, and these effects are not generally identified using standard toxicology tests (e.g., physiological responses and food-web mediated responses). The need for considering potential indirect effects as well sublethal direct effects was emphasized in the National Research Council review [28], as it recognized that not doing so may underestimate the true impacts of pesticides on ESA-listed species. While the importance of sublethal indirect effects on populations via trophic interactions has been well documented in field studies and mesocosm experiments [19], [52]–[54], formally incorporating them into risk assessments is an important step forward in ecotoxicology [16], [26], [28].

### Prey community resistance and recovery are key factors affecting susceptibility of salmon populations

While many researchers credit invertebrate communities with a capacity for “rapid” recovery, they often measure that in weeks to months. Our analyses indicate that even reductions in prey abundance for that “short” time can affect individual salmon and ultimately salmon populations, as they must feed throughout their freshwater residence. Resistance of the prey community is also important, but the greatest potential declines in λ occur when the prey community is slow to recover. This suggests indirect effects of pesticides on salmon are influenced by the prey community they are consuming. Prey recovery would be most rapid in communities with high connectivity, where colonizers can easily move in [55]–[57]. Typically these would be in less disturbed watersheds. However, even in less disturbed areas recovery may take weeks to months if habitats are relatively isolated, such as small tributaries and off-channel habitats, or if the community is dominated by univoltine taxa. Aquatic communities in small tributaries within an agricultural or urban watershed, for example, may have few if any sources of colonists and prey recovery may therefore be delayed because of a watershed’s position within a landscape.

### The frequency, duration & timing of exposures are important in determining effects on salmon population growth rates

Repeated exposures, even short in duration, produced particularly large reductions in salmon population growth rates. In addition, exposures that occur throughout the salmon growth period can have an equal or greater impact than isolated exposures early in the season. It may be possible to assess how frequently a habitat is exposed, but the long-term effects of multiple exposures on salmon also depend on the dynamics of the prey community (e.g., Figure 7). Salmon feeding in habitats with prey communities that are especially sensitive and slow to recover will be disproportionately impacted by repeated exposures.

### Data gaps and limitations of the model

As with all models, our analyses are limited by the data available. Parameters that are included, such as the prey recovery rates and the floor, were based on relevant literature values (e.g., [21], [41], [43]) but were not necessarily derived from experiments with pesticides in salmon habitats. For instance, we assume prey communities have a consistent sensitivity and recovery rate, but it is unlikely that after recurrent pesticide exposure, invertebrate communities would be as resilient as they were when first disturbed [58], [59]. Cuffney et al. [41] found that repeated seasonal pesticide pulses resulted in additional losses overall (effectively a lower prey floor with each pulse). Even though we assume all prey may be susceptible to repeated pulses, the model may underestimate effects on salmon population growth rates by not accounting for declines in resilience with repeated exposures. Likewise, macroinvertebrate toxicity values were primarily from 48 – 96-hour laboratory tests, and are not necessarily representative of field conditions. These represent data gaps that could be addressed with targeted research on the sensitivity and dynamics of aquatic invertebrate communities following chemical exposures.

Lack of data also limited which relationships were included in the model. For instance, the model incorporates time explicitly, but how organisms respond in space is not defined. While we assume 100% of a salmon population and its critical habitat are exposed to a particular concentration, monitoring data suggest it is more likely that exposures are patchy across a landscape [1], [60], with some fish and invertebrates affected more than others as they develop and move among habitats. Given the frequency and extent at which pesticides are detected, there may be few if any refuges in developed watersheds. Environmental data indicate that not only are pesticides detected at concentrations that exceed individual aquatic–life benchmarks [1], they are often detected multiple times throughout the year and in mixtures with other pesticides. For instance, Werner et al. [2] demonstrated how exposures can be recurrent yet patchy in their survey of 24 sites in the Sacramento and San Joaquin River Delta. They found nearly 10% of 400 water samples scattered across these sites over two years were toxic to the zooplankton *Ceriodaphnia dubia* [2], indicating a range of aquatic habitats may be exposed at some point in any one year. By not including spatial dynamics in exposure scenarios, the model may overestimate potential effects. Alternatively, assuming prey populations would be exposed to discrete exposures and then be able to recover may be unrealistic and cause the model to underestimate long-term effects.

Potential effects of pesticides on additional trophic levels and in combination with other stressors were also not included in the model due to lack of data. For instance, other than discrete pesticide exposures, the model does not include additional stressors (e.g., thermal stress, loss of shelter, additional pesticides, metals) that may additionally or synergistically impact fish and their prey [10], [29], [61], [62]. Furthermore, the model does not include effects on other trophic levels (e.g., primary producers) or ecosystem processes (e.g., organic matter processing, [41]).

### Future applications

Integrating potential direct and indirect effects of pesticide exposures in this model has improved our ability to distinguish and compare their relative importance under different environmental conditions. As additional data are available and relationships are better defined, this and similar population models evaluating effects of pesticides on ESA-listed species will be improved [25]. Model analyses are an important component of risk assessments (e.g., NOAA’s Biological Opinions [17], [18]), providing a transparent framework linking exposure and biological effects. Targeted pesticide monitoring and concurrent toxicity testing are critical for assessing risk of exposure and the toxicity of water and sediments [2], [15], [48], [63], but we also need to better understand how exposures affect prey densities and salmon feeding behavior in the field. By formally including the indirect effects on salmon via their prey and varying the prey community’s resistance and rate of recovery [26], the model helps determine which parameters influence salmon populations most and identifies empirical information needed to expand our understanding of community dynamics acting in these habitats.

## Supporting Information

Figure S1 **Probability plot of EC50 values for aquatic invertebrate species exposed to carbaryl.**
(TIF)

Supporting Information S1 **Prey sensitivity values.**
(DOCX)
