# Supplementary material for: A Modeled Comparison of Direct and Food Web-Mediated Impacts of Common Pesticides on Pacific Salmon
Source: PLoS One. 2014 Mar 31;9(3):e92436. doi: 10.1371/journal.pone.0092436 (PMC3970969; doi:10.1371/journal.pone.0092436)
Supplement: Supporting Information S1 — Prey sensitivity values. (DOCX) [file pone.0092436.s002.docx]

**Supporting Information S1. Prey sensitivity values**

The EC_50_ values for salmon prey sensitivity relied on previous analyses performed to assess the risks of diazinon, chlorpyrifos, and carbaryl to salmon and their prey ([[1](#_ENREF_1), [2](#_ENREF_2)]). While similar methods were used, some differences exist. The methods used for all three pesticides are summarized here. Details are available in NMFS (2008) for diazinon and chlorpyrifos and NMFS (2009) for carbaryl. To provide analyses that were consistent with those in the NMFS Biological Opinions, the EC_50_ values that were derived for those analyses were used here.

For each pesticide considered, available toxicological data were identified and used to determine an EC_50_ to represent salmon prey sensitivity. A search was completed using the EPA’s Ecotox database (http://cfpub.epa.gov/ecotox/), and the following criteria were used to determine which reported effect concentrations were included in the final analysis. The first criteria included data from studies on taxa that are known to be salmonid prey (or are functionally similar to salmonid prey); these include a diverse group of fresh and saltwater crustaceans, aquatic insects and worms. An exposure criteria selected studies with exposures of at least 24 hours (h) and not more than 96 h for inclusion. Finally, studies reporting invertebrate LC_50_s and EC_50_s in which mortality or immobilization of invertebrates was the recorded endpoint were included; the term “EC_50_” is used here to describe all of these included data. Data derived for sublethal endpoints (*e.g.*, growth or reproduction) were not included. If specific data were represented more than once in the Ecotox output, duplicates were eliminated. Additionally, literature searches identified data from several recent peer-reviewed studies that are not yet included in the Ecotox data base, but report effect concentrations that caused mortality, which were also included.

For diazinon, the median value based on the Ecotox database review was used as the EC_50_ value in the model analyses. For chlorpyrifos, the median value from the Ecotox database review (1.7 µg/L) was calculated and considered, but a similar, field-derived value (2.3 µg/L) was used instead. This latter value was considered more representative of potential effects on salmonid prey because it was derived from field experiments in which diverse aquatic invertebrate communities were exposed to various concentrations of chlorpyrifos ([[3](#_ENREF_3)]). Using data from a mesocosm study directly incorporates toxicity as well as community dynamics for a range of invertebrate species. Using original data from the authors (Paul van den Brink, personal communication), the relative abundances of taxa known to be salmonid prey (or functionally similar to salmonid prey) were calculated (i.e., sum of the abundances of 14 taxa at 7 days post-exposure divided by pre-treatment abundances). This data set was used because it allowed us to calculate an EC_50_ and slope for an assemblage of representative prey exposed to a relevant range of chlorpyrifos concentrations in replicated outdoor mesocosms. The calculated EC_50_ (2.3 µg/L) is similar to other published values (laboratory 96-hr EC_50_ for invertebrates range from 0.2 – 2.7 µg/L) ([[4](#_ENREF_4)]), and is consistent with its use as an insecticide.

For carbaryl, toxicity data from the Ecotox database were used, but a different method was employed to determine a single representative EC_50_ value [[2](#_ENREF_2)]. The method used in the NMFS Biological Opinion on carbaryl (2009) describes how available survival EC_50_ data were plotted using a cumulative probability distribution. The data were also plotted for the species without taking the geometric means. The distributions of individual species EC_50_s and the geometric means of EC_50_s grouped by taxa were analyzed to estimate the 50th, 10th, and 5th percentiles. Specifically, a probability plot was used to graph the EC_50_ concentrations normalized to a normal probability distribution (Figure S1). The X axis is scaled in probability (between zero and 100%) and shows the percentage of all of the individual data points whose value is less than the data point. The Y axis displays the range of the EC_50_ data on a log scale. The results of a linear regression of the log-transformed concentrations are shown and highlight the lognormal distribution of the data. In the regression equation, the normsinv function returns the inverse of the standard normal cumulative distribution. The standard normal distribution has a mean of zero and a standard deviation of one. For example, given a percentile value of 50 (*i.e.,* a probability of 0.5), normsinv(50) returns a value of zero. The plots and regressions were performed using KaleidaGraph 4.03 (Synergy Software).

The 10th percentile for carbaryl (4.33 μg/L), rather than the 50^th^ percentile (69.53 μg/L), was used as input for the population growth model exercises. The 10th percentile is a reasonable selection because the data included in the meta-analysis were limited to concentrations that caused mortality or immobilization within a short period of time (1-4 days). A growing number of studies on a variety of insecticides have reported that concentrations well below LC_50_s can cause delayed mortality or sublethal effects that may scale up to affect aquatic invertebrate populations, especially in scenarios with multiple exposures and/or other stressors. Evidence for ecologically significant sublethal or delayed effects to aquatic invertebrates includes reduced growth rates [[5](#_ENREF_5), [6](#_ENREF_6)], altered behavior [[7](#_ENREF_7)], reduced emergence [[7](#_ENREF_7), [8](#_ENREF_8)] reduced reproduction [[6](#_ENREF_6), [9](#_ENREF_9)], and reduced predator defenses [[7](#_ENREF_7), [10](#_ENREF_10)]. The selection of the 10th percentile is also a reasonable choice in keeping with general risk management practices of protecting the aquatic community. The standard procedure the U.S. EPA Office of Water uses in establishing Aquatic Life Criteria is to protect to the 5th percentile of a species sensitivity distribution that includes all genera of aquatic species for which valid data are available. If the 50^th^ percentile value were used, the overall assessment of the relative importance of direct and indirect effects would not change because the prey EC_50_ (69.53 μg/L) would remain considerably lower than the salmon AChE EC_50_ (145.8 μg/L).

References for supporting information

1. NMFS (2008) Endangered Species Act section 7 consultation: Environmental Protection Agency registration of pesticides containing chlorpyrifos, diazinon and malathion. Biological Opinion. Silver Spring, MD USA.

2. NMFS (2009) Endangered Species Act section 7 consultation: Environmental Protection Agency registration of pesticides containing carbaryl, carbofuran and methomyl. Biological opinion. Silver Spring, MD USA.

3. Van den Brink PJ, Van Wijngaarden RPA, Lucassen WGH, Brock TCM and Leeuwangh P (1996) Effects of the insecticide Dursban(R) 4E (active ingredient chlorpyrifos) in outdoor experimental ditches: II. Invertebrate community responses and recovery. Environmental Toxicology and Chemistry 15: 1143-1153.

4. Van Wijngaarden RPA, Van den Brink PJ, Crum SJH, Voshaar JHO, Brock TCM, et al. (1996) Effects of the insecticide Dursban(R) 4E (active ingredient chlorpyrifos) in outdoor experimental ditches: I. Comparison of short-term toxicity between the laboratory and the field. Environmental Toxicology and Chemistry 15: 1133-1142.

5. Schulz R and Liess M (2000) Toxicity of fenvalerate to caddisfly larvae: chronic effects of 1-vs 10-h pulse-exposure with constant doses. Chemosphere 41: 1511-1517.

6. Forbes VE and Cold A (2005) Effects of the pyrethroid esfenvalerate on life-cycle traits and population dynamics of Chironomus riparius - Importance of exposure scenario. Environmental Toxicology and Chemistry 24: 78-86.

7. Johnson KR, Jepson PC and Jenkins JJ (2008) Esfenvalerate-induced case-abandonment in the larvae of the caddisfly (Brachycentrus americanus). Environmental Toxicology and Chemistry 27: 397-403.

8. Schulz R and Liess M (2001) Acute and chronic effects of particle-associated fenvalerate on stream macroinvertebrates: A runoff simulation study using outdoor microcosms. Archives of Environmental Contamination and Toxicology 40: 481-488.

9. Cold A and Forbes VE (2004) Consequences of a short pulse of pesticide exposure for survival and reproduction of Gammarus pulex. Aquatic Toxicology 67: 287-299.

10. Sakamoto M, Chang KH and Hanazato T (2006) Inhibition of development of anti-predator morphology in the small cladoceran Bosmina by an insecticide: impact of an anthropogenic chemical on prey-predator interactions. Freshwater Biology 51: 1974-1983.

**Figure S1. Probability plot of EC50 values for aquatic invertebrate species exposed to carbaryl.**

Probability plot for carbaryl showing the distribution of the geometric means of the EC_50_s for each aquatic invertebrate species. The straight line shows the result of a linear regression. In the regression equation, the normsinv() function returns the inverse of the standard normal cumulative distribution.
